# Mechanism underlying the negative inotropic effect in rat left ventricle in hyperthermia: the role of TRPV1

**DOI:** 10.1186/s12576-020-00734-5

**Published:** 2020-02-05

**Authors:** Koji Obata, Hironobu Morita, Miyako Takaki

**Affiliations:** Department of Physiology, Gifu University Graduate School of Medicine, 1-1 Yanagido, Gifu, 501-1194 Japan

**Keywords:** TRPV1, LV contractility, Excitation–contraction coupling, Oxygen consumption, Hyperthermia

## Abstract

We have previously reported that the negative inotropic effects of hyperthermia (42 °C) on left ventricular (LV) mechanoenergetics using the excised, cross-circulated rat heart model. Here, we investigated the role of TRPV1 on LV mechanoenergetics in hyperthermia. We analyzed the LV end-systolic pressure–volume relation (ESPVR) and the linear relation between the myocardial oxygen consumption per beat (VO_2_) and the systolic pressure–volume area (PVA; a total mechanical energy per beat) during infusion of capsazepine (CPZ) in hyperthermia, or capsaicin (Cap) under 300 bpm pacing. LV ESP decreased in each LV volume and the resultant downward-shift of LV ESPVR was suppressed by CPZ infusion in hyperthermia-hearts. In Cap-treated hearts, LV ESPVR shifted downward from the control ESPVR, similar to hyperthermia-hearts. The slopes of VO_2_–PVA relationship were unchanged. The VO_2_ intercepts in hyperthermia-hearts did not decrease because of decreased E–C coupling VO_2_, and inversely increased basal metabolic VO_2_, which was suppressed by CPZ, though the VO_2_ intercepts in Cap-treated hearts significantly decreased. The levels of phosphorylated phospholamban at serine 16 decreased significantly in hyperthermia-hearts, as well as Cap-treated hearts. These results indicate that a Cap-induced decrease in the LV contractility, like in cases of hyperthermia, are due to the down-regulation of the total calcium handling in E–C coupling, suggesting that negative inotropic effect in hyperthermia-heart is, at least in part, mediated through TRPV1 signaling pathway.

## Background

Myocardial temperature sensitivity affects cardiac contractility following energy metabolism. In addition, the cardiac Troponin I is frequently elevated in patients with heat-related illnesses during a heat wave, which indicates myocardial damage [1]. Others studies, including our own, have previously reported that elevated cardiac temperature decreases left ventricular (LV) contractility and energy consumption, mechanoenergetics, in cardiac muscle strip and hearts isolated from rats, rabbits, or dogs [2–6]. Recently, we have shown that negative inotropic effect in hyperthermia (42 °C) is caused by a decrease in calcium (Ca^2+^) handling in excitation–contraction (E–C) coupling during which sarcoplasmic reticulum (SR) Ca^2+^-ATPase (SERCA) activity was suppressed due to phospholamban phosphorylation inhibition [5]. However, it remains unknown how the heart senses the hyperthermia conditions, and transmits the information to signal transduction pathway, which controls LV mechanoenergetics.

Transient receptor potential vanilloid 1 (TRPV1) is a nonselective cation channel that may be activated by a wide variety of exogenous and endogenous physical and chemical stimuli, such as pH, capsaicin (Cap), or temperatures above 43 °C (109 °F). Capsazepine (CPZ) is a competitive antagonist of TRPV1 which blocks the Cap-induced Ca^2+^ influx in sensory nerves. Cap-sensitive sensory nerves are widely distributed in the cardiovascular system, including in the heart, kidneys, and blood vessels [7–9]. Previous studies have reported a cardioprotective role for TRPV1 in myocardial ischemia and reperfusion injury [9, 10], in addition to attenuating cardiac hypertrophy [9, 11–14]. Thus, it is possible that TRPV1 works directly as a sensor for cardiac hyperthermia conditions and controls cardiac contractility and energy metabolism.

Ca^2+^ is also a key player in E–C coupling. SERCA is a Ca^2+^-ATPase which plays a major role on Ca^2+^ handling in E–C coupling. We previously reported that elevated cardiac temperature directly induces a negative inotropic action due to suppression of SERCA activity, owing to decreased phosphorylation of phospholamban (PLB) in Ca^2+^ handling without affecting neuro-, and/or humoral factors [5]. The increase in SERCA activity is elicited by phosphorylation of PLB at Ser^16^ by protein kinase A (PKA) and/or Thr^17^ by calmodulin-dependent protein kinase II (CaMK II) [15]. Previous studies demonstrated that gingerol, a TRPV1 agonists [16], activates Ca^2+^ pumping in skeletal and cardiac SR and ameliorates diabetes mellitus-induced diastolic dysfunction in isolated myocardium, suggesting that the activation of TRPV1 can increase SERCA activity and improve the diastolic function in hearts [17, 18]. On the other hand, the LV relaxation also determines the rate of dissociation in cross-bridge cycling and the number of myosin heads interacting with the thin filament (actin) related to myosin ATPase activity. In fact, we have previously shown that the logistic time constant is significantly shortened in hyperthermia [5], which may indicate the acceleration of relaxation by increasing myosin ATPase activity, which related to the increased TRPV1 activity in hyperthermia. Thus, it is possible that TRPV1 agonist or its antagonist exerts cardioprotective effects against damages from heatstroke or severe fevers.

The aim of the present study is to clarify the direct effects of TRPV1 activation on cardiac function and energy metabolism. We investigated the role of TRPV1 in hyperthermia by treatment with CPZ or Cap on LV myocardial mechanoenergetics using the excised, cross-circulated rat heart model to reveal whether TRPV1 acts as molecular micro-thermometers in cardiomyocytes.

## Methods

### Experimental animals

Our investigations were in accordance with the *Guide for the Care and Use of Laboratory Animals* published by the US National Institutes of Health (NIH Publication No. 85-23, revised 1996), and reviewed and approved by the Animal Research Committee of Gifu University (Gifu, Japan). Three male Wistar rats weighing 464 ± 57.3 g were used in each experiment. The largest rat in weight was used as blood supplier. The middle-sized rat was used as metabolic supporter for the excised heart. The smallest rat was used as heart donor in excised cross-circulation rat heart preparation.

### Excised cross-circulated rat heart model

We used the excised, cross-circulated rat heart preparation as previously reported [19–25]; we have also described the same in detail in the Additional file 1: Figure S1.

### Data analysis

We analyzed the obtained data in excised, cross-circulated rat heart preparations as previously reported [19–25], and also described it in detail in Additional file 1: Fig. S2A, B.

### Analyses of one-beat LV pressure–time curve by logistic function

We analyzed “logistic” time constant from respective best-fit functions to one-beat LV pressure–time curve at midrange LV volume (mLVV) during relaxation, with our proposed “logistic function” to evaluate LV end-diastolic relaxation rate or lusitropism [26] at 37 °C (*n* = 8–10 experiments, i.e., excised hearts), 42 °C (*n* = 10), 42 °C + CPZ (*n* = 10), and Cap (*n* = 8).

### Experimental protocol

LV volume (LVV) changes were measured by adjusting the intra-balloon water volume with a syringe in 0.025-mL steps between 0.08 mL and 0.23 mL (5–6 different volumes) (volume-loading run: vol-run) in the presence or absence of CPZ or Cap at 37 °C or 42 °C (Additional file 1: Figure S1). In every vol-run, steady state (where LVP, coronary arteriovenous O_2_ content difference (AVO_2_D), and CBF were stable), was reached 2–3 min after changing LVV. Cardiac arrest was induced by infusing KCl (0.5 mol/L) into the coronary perfusion tubing at a constant rate (5–10 mL/h) with a syringe pump in the presence or absence of CPZ or Cap at 37 °C or 42 °C, to measure the basal metabolic O_2_ consumption. KCl-cardiac arrest was adjusted to abolish electrical excitation while monitoring ventricular electrocardiograms, but not to generate any KCl-induced constrictions of coronary vessels. VO_2_ and PVA data were obtained by minimal volume loading to avoid volume-loading effects, if any, on VO_2_ data.

CPZ and Cap were purchased from Wako Pure Chemical Industries, Ltd. (Osaka, Japan). Cap was dissolved as previously reported [27]. CPZ was dissolved in EtOH at a concentration of 37.7 mg/mL and was diluted to 377 µg/mL in 1% EtOH. We confirmed no effect on LV mechanoenergetics with 1% EtOH as a vehicle, because final EtOH concentration in blood was very low about 0.0002–0.005%. CPZ [final concentration, 1–2 µg/mL (approximately 2–5 µM) at a coronary flow of 2–5 mL/min] was perfused at 5–10 µL/min for 15 min before, for 40–45 min with micro-syringe pump during volume loading (vol)-run and KCl-cardiac arrest (Additional file 1: Figure S1). Cap [final concentration, 20–500 ng/mL (approximately 0.2–4 µM) at a coronary flow of 2–5 mL/min] was perfused at 1–20 µL/min for 15 min before, for 40–45 min with micro-syringe pump during vol-run, inotropism (ino)-run, and KCl-cardiac arrest (Additional file 1: Figure S1).

All data were measured and sampled at 1 kHz for 5 − 10 s and averaged using a PowerLab unit and LabChart software (AD Instruments, Bella Vista, NSW, Australia).

### Immunoblotting analysis for PLB, phosphorylated-PLB (p-PLB), and GAPDH

Immunoblotting analysis was performed as previously reported [5, 21, 28, 29]. In brief, total proteins were purified from the LV free wall of each frozen heart and stored at − 80 °C after the mechanoenergetic studies. Proteins (50 µg/lane) were separated on a 15% sodium dodecyl sulfate (SDS)-polyacrylamide gels in a minigel apparatus (Mini-PROTEAN II, Bio-Rad Laboratories, Inc., CA) and transferred to polyvinylidene difluoride (PVDF) membranes. The membranes were blocked (4% Block Ace, Dainippon Pharmaceutical Co., Osaka, Japan) and then incubated with primary antibody against anti-PLB antibody (1:1000 dilution, Upstate Biotechnology, Inc., MA), p-PLB at serine 16 residue (p-PLB^Ser16^, Abcam, Cambridge, UK), and p-PLB at threonine 17 residue (p-PLB^Thr17^, Badrilla Ltd, Leeds, UK). Detection was performed by the luminescence method (ECL Western blotting detection kit, GE Healthcare Japan, Tokyo, Japan) with peroxidase-linked anti-mouse IgG (1:5000 dilution) or peroxidase-linked anti-rabbit IgG (1:5000). The bands were normalized to anti-GAPDH antibody (Cell Signaling Technology Inc. MA) to confirm equal loading of samples. Band intensity was analyzed with ImageJ/Fiji software.

### Statistics

Multiple comparisons were performed by one-way analysis of variance (ANOVA) with post hoc Bonferroni’s test. Alternatively, comparison of unpaired individual values was performed by unpaired *t* test. A value of *p* < 0.05 was considered statistically significant. All data are expressed as the mean ± S.D.

## Results

### Inhibition of hyperthermia-induced negative inotropic effect by CPZ

**Fig. 1:**
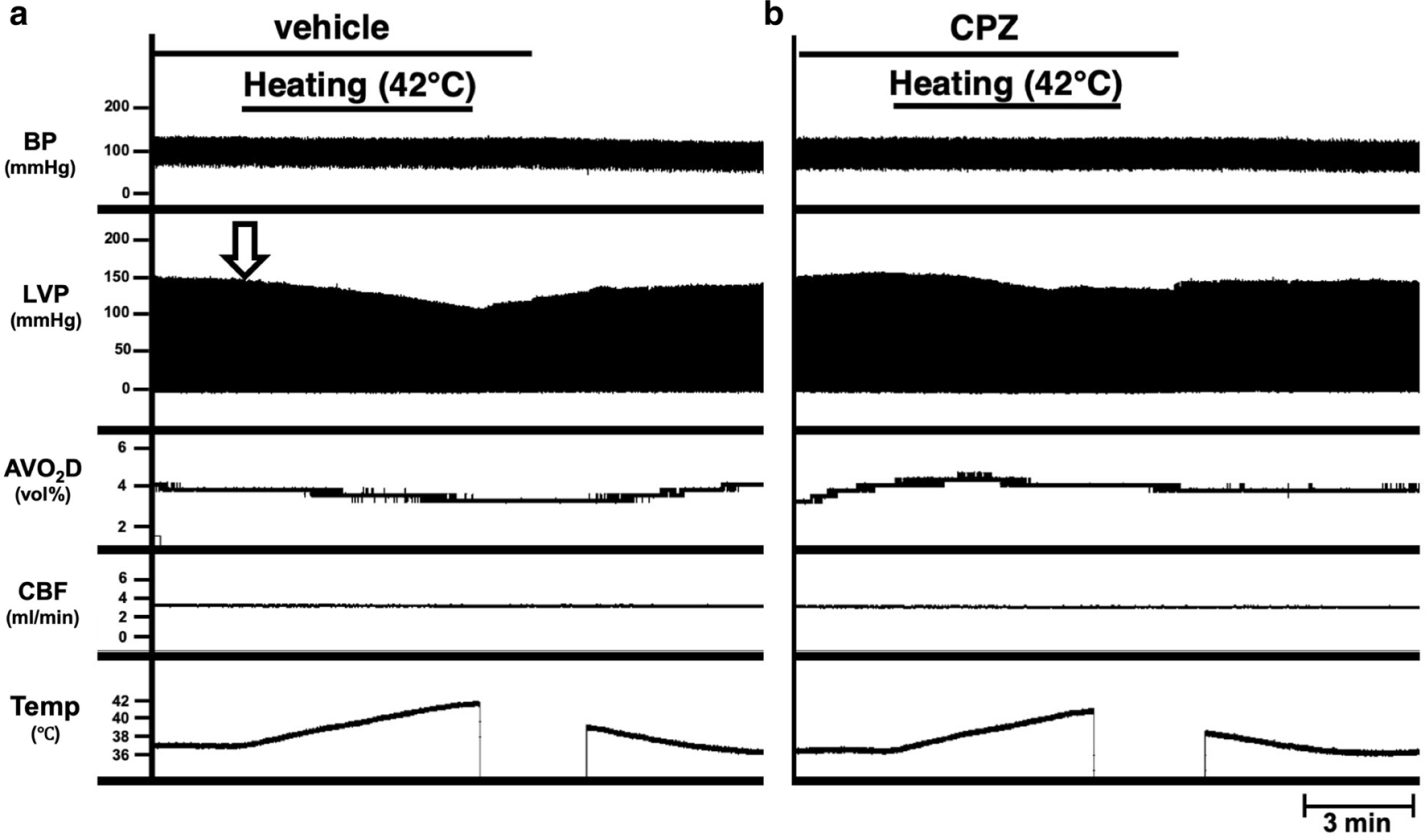
Effects of capsazepine (CPZ) in simultaneous recordings of blood pressure of a support rat; perfusion pressure (BP), left ventricular pressure (LVP), arteriovenous oxygen content difference (AVO_2_D), coronary blood flow (CBF), and circulatory blood temperature (Temp) at midrange left ventricular volume (mLVV) during heating at 42 °C in the absence (**a**) or presence of CPZ (**b**) in an excised, cross-circulated rat heart preparation. The open arrow in the left panel (**a**) indicates that the value of end-systolic pressure (ESP) on LVP gradually decreased from 146 to 110 mmHg until 42 °C after onset of heating, which was partially suppressed by the infusion of CPZ (approximately 1.23 µg/mL in blood) (**b**)

We previously reported that LV end-systolic pressure (ESP) decreased and thus LV end-systolic pressure–volume relation (ESPVR) shifted downward in hyperthermia conditions at 42 °C [5]. First, we used a TRPV1 antagonist, CPZ to clarify the role of TRPV1 as a molecular thermometer on negative inotropic effect in hyperthermia-hearts. The LV ESP gradually decreased from 146 to 110 mmHg up to 42 °C after onset of heating and returned to the original value after heating stopped (Fig. 1a). This ESP decrease was suppressed by the infusion of CPZ (approximately 1.23 µg/mL in blood) (Fig. 1b). The LV end-diastolic pressure (EDP) was maintained during both heating and CPZ-treatment around zero mmHg (Fig. 1a, b). According to the decrease of LVP in hyperthermia, the AVO_2_D also slightly decreased. The decrease in AVO_2_D was also completely inhibited by CPZ. CBF did not change when the temperature of perfusion blood was increased in the presence or absence of CPZ (Fig. 1). This result indicates that the negative inotropic effect in hyperthermia is inhibited by CPZ, suggesting the possibility for an association with the TRPV1 signaling pathway.

### LV mechanoenergetics during CPZ infusion in hyperthermia

**Fig. 2:**
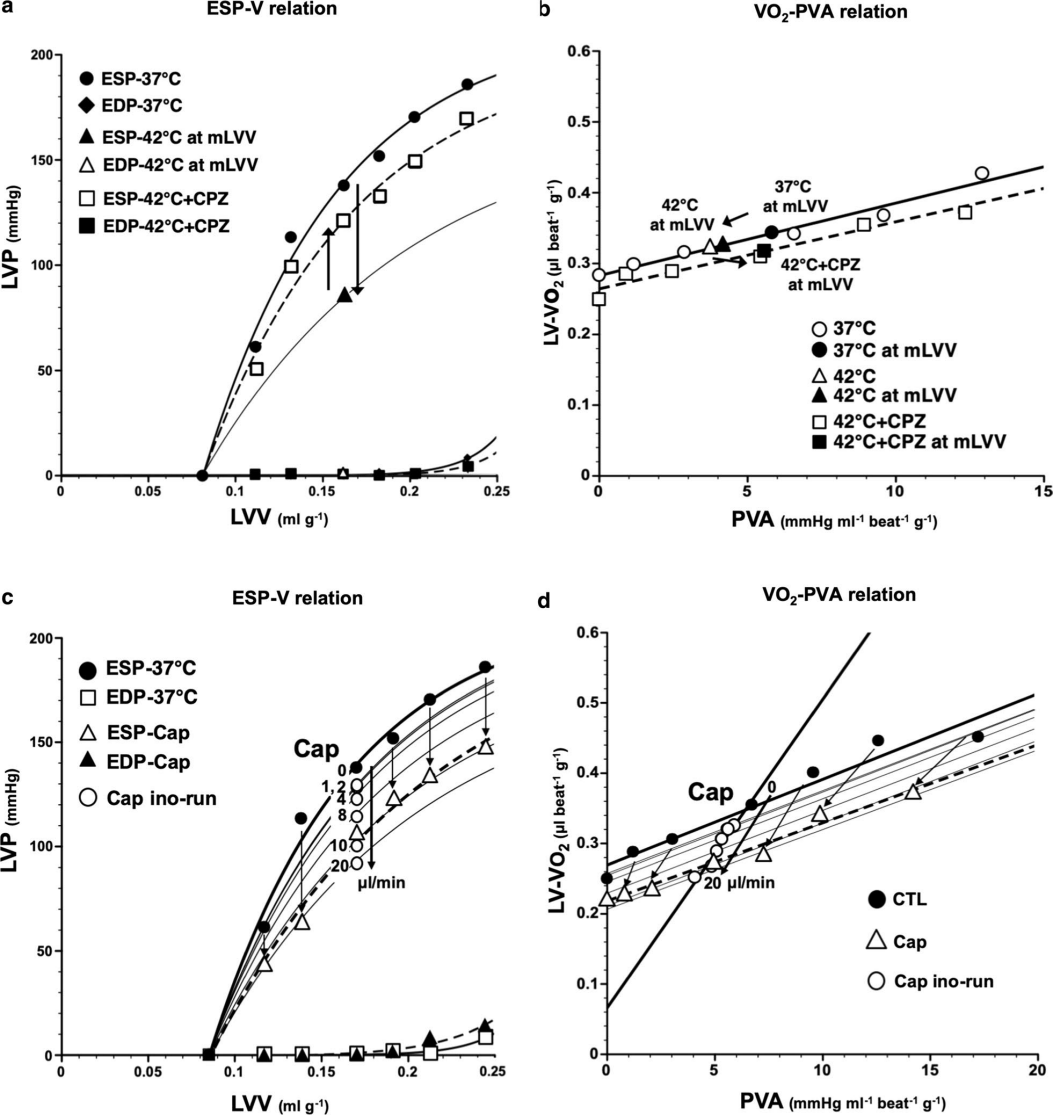
Representative data of LV end-systolic pressure–volume relation (ESPVR), LV end-diastolic pressure–volume relation (EDPVR), and myocardial oxygen consumption per beat (VO_2_)-systolic pressure–volume area (PVA) relation in the presence of CPZ (1.59 µg/mL in blood) during 42 °C (**a** and **b**, dotted lines) and in the presence of capsaicin (Cap) (0–461 ng/mL in blood) at 37 °C (**c** and **d**, dotted lines), respectively. The arrows in panel A indicate that the decrease in LV ESP at mLVV at 42 °C (solid triangle) was partially inhibited by CPZ (open square). The fine line indicates the estimated LV ESPVR at 42 °C. Thus, the VO_2_–PVA data point at mLVV in CPZ-treated heart at 42 °C (solid square) shifted right-downward from that in hyperthermia-heart (solid triangle), which left-downward shifted data point from it at 37 °C (solid circle) (**b**). On the other hand, the LV ESPVRs in Cap-treated heart shifted downward (**c**) and each PVA and VO_2_ values (open triangles) at each LVV during Cap infusion (230 ng/mL in blood) was smaller than each control value (solid circles), and the VO_2_–PVA linear relations during Cap infusion shifted downward; VO_2_-intercept values decreased without changes in the slope (**d**). The open circles indicate that Cap dose-dependently decreased the LV ESP and thus shifted in parallel estimated VO_2_–PVA relation according to stepwise elevation of the Cap infusion rate (0, 1, 2, 4, 6, 8, 10, 20 µL/mL) with infusion pump (**c** and **d**). The fine lines indicate the estimated LV ESPVRs and VO_2_–PVA linear relations at various Cap infusion rates **c** and **d**)

**Fig. 3:**
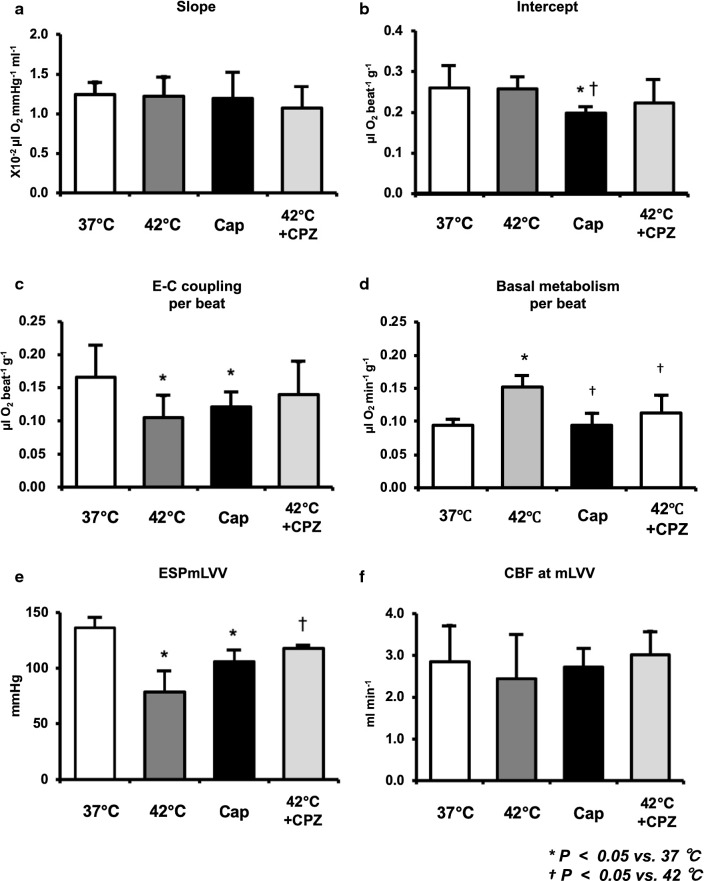
Comparison of the mean slope (oxygen cost of PVA; **a**), mean VO_2_ intercept (PVA-independent VO_2_; **b**), mean VO_2_ consumed in E–C coupling (**c**), mean basal metabolic VO_2_ (**d**), mean ESP at mLVV (**e**), and mean CBF at mLVV (**f**) in the presence or absence of Cap or CPZ at 37 °C or 42 °C. Group data are mean ± SD. *p <* 0.05 vs. 37* *°C,*^†^*p *<* 0.05 vs. 42* *°C.* The mean slope and mean CBF did not change in Cap- or CPZ-treated hearts during 37 °C or 42 °C (**a**, **f**). The mean VO_2_ intercepts in Cap-treated hearts were significantly lower than that at 37 °C (**b**), which was due to the decrease in mean VO_2_ consumed in E–C coupling (**c**) without changing mean basal metabolic VO_2_ (**d**). The decrease in the mean VO_2_ for E–C coupling and the increase of mean basal metabolic VO_2_ in hyperthermia-hearts without changing mean VO_2_ intercepts was inhibited by CPZ-treatment (**c**, **d**). The mean ESP at mLVV in hyperthermia- and Cap-treated hearts were significantly lower than that during 37 °C (**e**). The decrease of mean ESP at mLVV in hyperthermia-hearts significantly inhibited by CPZ-treatment (**e**)

The decrease in ESP at mLVV from 37 to 42 °C was markedly suppressed by CPZ-treatment in the same heart, though not completely (Fig. 2a). Thus, the decrease of mean ESP at mLVV in hyperthermia-hearts was significantly inhibited by CPZ-treatment (Fig. 3e). We had already reported that the slope and intercept for VO_2_–PVA linear relation did not change in hyperthermia conditions, though ESPVR shifted downward from that in normothermia [5]. The VO_2_–PVA data point at mLVV in a CPZ-treated hyperthermia-heart shifted right-downward (indicated by a solid square) from that in a hyperthermia-heart (indicated by a solid triangle), which shifted left-downward from that in normothermia (indicated by a solid circle) (Fig. 2b). However, mean slopes and VO_2_ intercepts for VO_2_–PVA linear relations did not change in CPZ-treated hyperthermia-hearts (Fig. 3a, b). The decrease in mean VO_2_ for E–C coupling and the increase in mean basal metabolic VO_2_ in hyperthermia-hearts, without changing VO_2_ intercepts, were inhibited by CPZ-treatment (Fig. 3c, d, though not significant in C). The results suggest that CPZ, though not completely, inhibits hyperthermia-induced mechanoenergetics, suggesting that TRPV1 signaling pathway may inhibit the decrease of VO_2_ for E–C coupling and the increase in VO_2_ for basal metabolism in hyperthermia. Mean slope, intercept, VO_2_ for E–C coupling and basal metabolism, LV ESP and CBF at mLVV did not change in CPZ-treated heart at 37 °C (data not shown).

### LV mechanoenergetics during Cap infusion

An LV ESP-V data point shifted downward in a dose-dependent manner during Cap ino-run and, therefore, during Cap vol-run at 10 µL/min, LV ESPVR shifted downward (Fig. 2c) and mean ESP at mLVVs was significantly lower than that at 37 °C (Fig. 3e). LV EDPVR remained almost unchanged during the Cap vol-run (Fig. 2c). These results suggest that the hyperthermia-induced negative inotropic action was caused by the Cap-sensitive TRPV1 signaling pathway. The VO_2_–PVA linear relationship during the Cap vol-run shifted downward in parallel, suggesting that the mean VO_2_ intercept (PVA-independent VO_2_), composed of the VO_2_ for E–C coupling and basal metabolism, decreased significantly in Cap-treated hearts, unlike that in hyperthermia (Figs. 2d, 3b). The decline in mean VO_2_ intercept in Cap-treated hearts was caused by the decrease in VO_2_ consumed in E–C coupling without changing basal metabolic VO_2_ (Fig. 3c, d). The slopes which inversely means the efficiency for converting chemical energy into mechanical work did not alter in Cap-treated hearts like in hyperthermia (Figs. 2a, 3a). The results suggest that the effects of Cap on LV mechanoenergetics were somewhat different from the effects in hyperthermia, although both Cap and hyperthermia exerted negative inotropic effects. CBF did not change in Cap-treated hearts (Fig. 3f).

### Immunoblotting of PLB, p-PLB^Ser16^, and p-PLB^Thr17^ in Cap- or CPZ-treated hearts in normothermia or hyperthermia

**Fig. 4:**
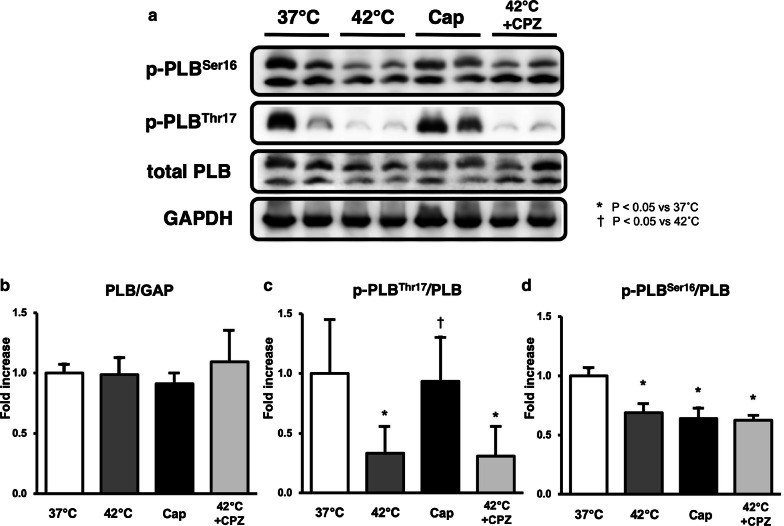
Western blot analysis of total phospholamban (PLB), phosphorylated phospholamban on Ser16 (p-PLB ^Ser16^) and Thr17 (p-PLB^Thr17^), and GAPDH in LV tissues of the presence or absence of Cap or CPZ at 37 °C or 42 °C. Representative data of total PLB, p-PLB ^Ser16^, and p-PLB^Thr17^ (**a**). Comparison of the mean protein levels of total PLB/GAP (**b**), p-PLB^Ser16^/PLB (**d**), and p-PLB^Thr17^/PLB (**c**). Values are mean ± SD of 5–6 LV tissues for each experimental group. *p < *0.05 vs. 32* *°C,*^†^*p *<* 0.05 vs. 42* *°C*

The phosphorylation of PLBs, especially p-PLB^Thr17^ was remarkably decreased in hyperthermia-hearts, but was unchanged in Cap-treated hearts (Fig. 4a, c). Conversely, mean levels of p-PLB^Ser16^ decreased significantly in hyperthermic and Cap-treated hearts (Fig. 4a, d). These results indicate that elevated cardiac temperature and Cap-treatment may regulate the phosphorylation (dephosphorylation) of the PLB signaling pathway. Interestingly, reduction in p-PLB^Thr17^ and p-PLB^Ser16^ in hyperthermic hearts was not inhibited by CPZ-treatment (Fig. 4c, d). The expression levels of PLB protein did not change in Cap-treated hearts at 37 °C or in CPZ-treated hearts at 37 °C or 42 °C (Fig. 4a, b). These results suggest that the decrease in VO_2_ for E–C coupling in both hyperthermic- and Cap-treated hearts is induced by a reduction in SERCA activity, which occurs as a result of decreased p-PLB.

### Logistic time constants during Cap in normothermia and CPZ in hyperthermia

**Fig. 5:**
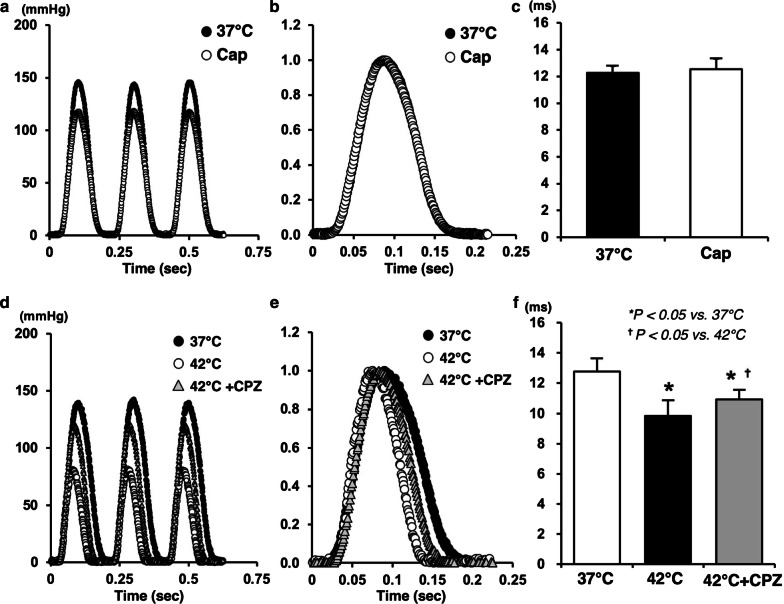
Representative data of LV pressure–time curves (**a**, **d**), normalized of LV pressure–time curves (**b**, **e**), and logistic time constants (**c**, **f**) at mLVV in the presence or absence of Cap or CPZ during 37 °C or 42 °C. Values are mean ± SD of six LV pressure–time curves at mLVV for each experimental group (**c**, **d**). *p < *0.05 vs. 37* *°C,*^†^*p *<* 0.05 vs. 42* *°C*

Mean duration of LV relaxation time significantly decreased in hyperthermia-hearts, but did not change in Cap-treated hearts (Fig. 5c, f). This decrease in hyperthermia is associated with temperature-dependent myosin ATPase activity in cross-bridge cycling, whereas Cap is not likely to act on it directly. The decrease in LV relaxation time in hyperthermia was partially inhibited in the CPZ-treated hearts (Fig. 5f). These results suggest that cross-bridge dissociation and/or the acceleration of intracellular Ca^2+^ uptake in SR through SERCA2a is sped-up via the TRPV1 signaling pathway.

## Discussion

In the present study, we demonstrated that the TRPV1 antagonist, CPZ, inhibits the negative inotropic action in hyperthermia-hearts. We also show that the TRPV1 agonist, Cap also induces negative inotropic effects with the decrease of VO_2_ for E–C coupling like in hyperthermia-hearts. Mechanoenergetic analysis revealed that the VO_2_–PVA slopes were not significantly different in hyperthermia-, CPZ-treated hyperthermia-, or Cap-treated hearts. The VO_2_ intercept of the VO_2_–PVA linear relation did not change in CPZ-treated hyperthermia-hearts, but the VO_2_ intercept in Cap-treated hearts decreased due to the decrease in VO_2_ for E–C coupling. E–C coupling VO_2_ decreased and basal metabolic VO_2_ increased in hyperthermia-hearts; however, the E–C coupling VO_2_ tended to increase and the basal metabolic VO_2_ significantly decreased in the CPZ-treated hyperthermia-hearts compared to hyperthermia-hearts. Western blotting analysis revealed that the ratio of p-PLB^Ser16^/PLB significantly decreased in both hyperthermia- and Cap-treated hearts, but the ratio of p-PLB^Thr17^/PLB remarkably decreased only in hyperthermia-hearts. Thus, we considered that the negative inotropic effects in hyperthermia-hearts could be caused by a decrease in Ca^2+^ handling due, at least in part, to Cap- and/or CPZ-sensitive TRPV1 signaling.

First, we examined whether the administration of the TRPV1 antagonist, CPZ, can inhibit the negative inotropism in hyperthermia-hearts using the excised, cross-circulated rat heart model (Additional file 1: Figure S1). As a result, CPZ suppressed the decrease of LV ESP in hyperthermia-heart (Figs. 1, 2a). These results astonished us, because previous studies reported that CPZ does not block acid- or heat-induced activation of TRPV1 in sensory nerves [30, 31]. Therefore, the inhibition of negative inotropism in CPZ-treated hyperthermia-hearts may be caused by blocking of other TRPV1 subtypes, or by the non-selective action of CPZ. We also found that the TRPV1 agonist, Cap, also shifted the LV ESPVR downward in a dose-dependent manner (Fig. 2c). Therefore, we considered that the negative inotropic effects in hyperthermia-hearts may be mediated through the TRPV1 signaling pathway. In fact, we previously reported that high-dose Cap induces negative inotropic effects on cardiac muscles [27].

TRPV1 is located on the cardiac sensory nerves and might function as a molecular sensor to detect tissue ischemia and activate cardiac nociceptors because a selective antagonist of TRPV1, iodoresiniferatoxin attenuates both bradykinin- and ischemia-induced firing of cardiac spinal afferent nerves [32, 33]. In contrast, Andrei et al. demonstrated that TRPV1 is functionally expressed in cardiac myocytes of adult mice and co-localizes at z-discs, costameres and intercalated discs [34]. Hurt et al. showed that TRPV1 localizes in the mitochondria of primary neonatal cardiomyocytes [10]. Therefore, TRPV1 in cardiomyocytes may have several subtypes and the roles that they may play is still up for debate. In the present study, we used the excised, cross-circulated rat heart model, which is a suitable for evaluating the direct effects of TRPV1 agonists or antagonists in hearts (cardiomyocytes).

The VO_2_–PVA slopes were not significantly different among hyperthermia-, hyperthermia CPZ-treated, or Cap-treated hearts, which means that the oxygen cost of PVA (i.e., the efficiency of chemo-mechanical energy transduction), was maintained regardless of the status of TRPV1 in the excised rat hearts. These results are consistent with previous observation in hyperthermia rat hearts [5], and Cap-treated or hyperthermia canine hearts [4, 6, 27].

We have previously shown that the VO_2_ intercept in hyperthermia-hearts did not change because of the decreased VO_2_ for E–C coupling and reversely increased basal metabolic VO_2_ [5]. We found that the VO_2_ intercept did not change in CPZ-treated hyperthermia hearts (Figs. 2b, 3b). However, the decrease in E–C coupling VO_2_ and the increase in the basal metabolic VO_2_ were significantly inhibited in CPZ-treated hyperthermia hearts (see Fig. 3c, d). However, the VO_2_ intercept decreased in Cap-treated hearts due to a decrease in VO_2_ for E–C coupling (Figs. 2d, 3b–d). Therefore, both hyperthermia and Cap-treatment can induce the decrease of VO_2_ for E–C coupling, which may be mediated by the activation of TRPV1 in cardiomyocytes. However, CPZ predominantly suppressed the increase of the basal metabolic VO_2_ in hyperthermia-hearts but Cap did not increase the basal metabolic VO_2_, unlike in hyperthermia-hearts. This means that the VO_2_ for basal metabolism may be affected by a hyperthermia- and CPZ-sensitive TRPV1, which is different from a Cap-sensitive TRPV1.

The VO_2_ for E–C coupling mainly means the energy consumption by SERCA2a for SR Ca^2+^ uptake in cardiomyocytes. SERCA2a plays a crucial role in diastolic function in heart. In the present study, the decrease of VO_2_ for E–C coupling in both hyperthermia- or Cap-treated hearts might be caused by a decline of amplitude in transient Ca^2+^, resulting in the negative inotropic effect. Previous studies reported that the amplitude of transient Ca^2+^ was significantly decreased by increasing temperatures from 37 to 40 °C in vitro in embryonic chick hearts [35]. However, the force development decreased under hyperthermic conditions (42 °C), with unchanged intracellular transient Ca^2+^ using rat isolated cardiac trabeculae [3]. It was concluded that the Ca^2+^ kinetics was accelerated, as a result, the time for myofilament activation reduced under hyperthermia.

Interestingly, the logistic time constant significantly shortened in hyperthermia, which was partially suppressed by CPZ-treatment, but remained unchanged by Cap-treatment (Fig. 5). Therefore, the effect of Cap or hyperthermia on the LV lusitropy was not similar despite both having negative inotropic effects. Thus, LV lusitropy might be, at least in part, mediated through TRPV1 signaling because CPZ partially inhibited the shortening of logistic time constant during hyperthermia. We previously reported that a possible mechanism for the negative inotropic effect in hyperthermic hearts could be considered to accelerate the rate of detachment in cross-bridge cycling and/or decreasing the number of myosin heads interacting with the thin filament (actin) due to increased myosin ATPase activity [5]. Myosin and actin interaction (i.e., cross-bridge cycling) might not be affected by a Cap-sensitive TRPV1 signaling pathway because Cap did not change the logistic time constant. Thus, the activity of myosin ATPase might be mainly dependent on temperature condition. The inhibition of shortened logistic time constant by CPZ might be caused by the decreased Ca^2+^ handling in E–C coupling rather than myosin and actin interaction. These results suggest that the negative inotropic action in Cap-treated hearts might be induced by a decrease in VO_2_ for E–C coupling as well as by the decline in amplitude in transient Ca^2+^ through TRPV1 signaling pathway. However, the LV diastolic function may be at least in part mediated through a hyperthermia- and CPZ-sensitive TRPV1, but may not be affected by a Cap-sensitive TRPV1. The VO_2_ for basal metabolism might be related to the different effects of TRPV1 in mitochondria of cardiomyocytes.

Phosphorylation of PLB (p-PLB) at either Ser^16^ by PKA, or Thr^17^ by CaMK II elevates SERCA activity (i.e., the acceleration of intracellular Ca^2+^ uptake to the SR) [15]. Therefore, the down-regulation of p-PLB (Fig. 4) indicates a decline in SERCA activity, which was supported by the decrease in VO_2_ for E–C coupling in both hyperthermic and Cap-treated hearts. The phosphorylation of PLB is the main determinant of β1-adrenergic responses. Although phosphorylation of Thr^17^ by CaMKII contributes to this effect, its role is subordinate to that of the PKA-dependent increase in cytosolic Ca^2+^ that is necessary to activate CaMKII [36]. Therefore, phosphorylation at Ser^16^ precedes that of Thr^17^ in hearts. A shift from p-PLB^Ser16^ to p-PLB^Thr17^ was observed under prolonged β1-adrenergic stimulation [37].

Here we demonstrate that the ratio of p-PLB^Thr17^/PLB decreases significantly in hyperthermic hearts—unlike in Cap-treated hearts—and that the ratio of p-PLB^Ser16^/PLB decreases significantly to the same degree in both the hyperthermia and Cap-treated hearts. Interestingly, CPZ did not suppress reduction in p-PLB^Thr17^ and p-PLB^Ser16^ (Fig. 4). These results suggest that the decrease in p-PLB^Ser16^ represents the down-regulation of PKA activity in both hyperthermic and Cap-treated hearts, and that the decrease in PLB^Thr17^ represents the down-regulation of CaMKII activity only in the hyperthermic hearts, which may be independent of a CPZ-sensitive signaling pathway. The inhibitory mechanisms of PKA and/or CaMKII via TRPV1 are unclear, but previous studies have reported a cardioprotective role for TRPV1 in myocardial ischemia and reperfusion injury [9, 10]. CaMKII-dependent phosphorylation of PLB has been linked to protective effects in both acidosis and ischemia/reperfusion [38]. Hyperthermia-sensitive TRPV1, but not Cap- and CPZ-sensitive TRPV1 may protect against myocardial acidosis and ischemia/reperfusion injury via CaMKII signaling pathway. Phosphorylation of PLB is also dependent on the activity of the type 1 phosphatase (PP1). Dephosphorylation of PLB reverses the activation of SERCA2a [39]. The PP1 activity is controlled by several kinases and phosphatases. TRPV1, but not CPZ-sensitive TRPV1, may contribute to activate these signaling pathways. Further investigation is needed to clarify the questions.

Although CPZ significantly inhibited the negative inotropic effect during hyperthermia, CPZ partially improved the decrease in VO_2_ for E–C coupling and did not improve the decrease in p-PLBs. The results suggest that CPZ did not completely recover Ca^2+^ handling in E–C coupling including SERCA2a activity. On the other hand, CPZ inhibited the shortening of the logistic time constant during hyperthermia despite no shortening of that during Cap-treatment. The reason is uncertain, but studies have reported that TRPV1 expressed in mouse skeletal muscle presents only at the SR membrane and functions as an SR Ca^2+^ leak channel [40]. Previous studies also reported that TRPV1 localizes at the z-discs, costameres, and intercalated discs [34] or at the mitochondria in cardiomyocytes [10]. Therefore, we hypothesize that a hyperthermia-sensitive, a Cap-sensitive, or a CPZ-sensitive TRPV1 subtypes may exist in cardiomyocytes and that these subtypes may have different localizations and functions. In fact, previous studies demonstrated that the pharmacological actions of capsaicin are elicited via TRPV1-independent mechanisms in many organs or cells except for heart (cardiomyocytes) [41–46]. Therefore, the present study would be the first to show the evidence for the TRPV1-independent action of capsaicin in cardiac mechanoenergetics.

## Conclusion

In conclusion, we have provided evidence that in hyperthermia-hearts TRPV1 plays an important role in negative inotropic action using the excised, cross-circulated rat heart model. CPZ inhibited the negative inotropic effects by improving Ca^2+^ handling and basal metabolism in hyperthermia-hearts. Both hyperthermia and Cap induced the negative inotropic action, which may relate to the decrease of SERCA activity due to the decline of p-PLB via TRPV1 signaling pathway. In clinical implication, we expect TRPV1 antagonists including CPZ may also exert cardioprotective effects against damages from heatstroke or severe fevers due to the inhibition of negative inotropism in hyperthermia conditions. We conclude that hyperthermia-induced negative inotropic action is mediated via TRPV1 which acts as a molecular micro-thermometer.

### Supplementary information

**Additional file 1.** Supplemental methods for the excised cross-circulated rat heart model and the data analysis. **Figure S1.** Schematic illustration of experimental setting for the excised blood-perfused rat heart. **Figure S2.** Schematic illustration of framework of ESPVR-VO2-PVA.

## Data Availability

The datasets used and/or analyzed during the current study are available from the corresponding author on reasonable request.

## Abbreviations

LV: Left ventricular
TRPV: Transient receptor potential vanilloid
ESP: End-systolic pressure
EDP: End-diastolic pressure
ESPVR: ESP–volume relation
EDPVR: EDP–volume relation
VO_2_: Myocardial oxygen consumption per beat
PVA: Pressure–volume area
Bpm: Beats per minute
CPZ: Capsazepine
Cap: Capsaicin
E–C: Excitation–contraction
PLB: Phospholamban
SR: Sarcoplasmic reticulum
PKA: Protein kinase A
CaMK II: Calmodulin-dependent protein kinase II
mLVV: Midrange LV volume
AVO_2_D: Arteriovenous O_2_ content difference
CBF: Coronary blood flow

## References

[1] Hausfater P, Doumenc B, Chopin S, Le Manach Y, Santin A, Dautheville S, Patzak A, Hericord P, Mégarbane B, Andronikof M, Terbaoui N, Riou B (2010). Elevation of cardiac troponin I during non-exertional heat-related illnesses in the context of a heat wave. Crit Care.

[2] Goto Y, Slinker BK, LeWinter MM (1991). Effect of coronary hyperemia on Emax and oxygen consumption in blood-perfused rabbit hearts Energetic consequences of Gregg’s phenomenon. Circ Res.

[3] Hiranandani N, Varian KD, Monasky MM, Janssen PM (2006). Frequency-dependent contractile response of isolated cardiac trabeculae under hypo-, normo-, and hyperthermia conditions. J Appl Physiol.

[4] Mikane T, Araki J, Suzuki S, Mizuno J, Shimizu J, Mohri S, Matsubara H, Hirakawa M, Ohe T, Suga H (1999). O_2_ cost of contractility but not of mechanical energy increases with temperature in canine left ventricle. Am J Physiol Heart Circ Physiol.

[5] Obata K, Takeshita D, Morita H, Takaki M (2018). Left ventricular mechanoenergetics in excised, cross-circulated rat hearts under hypo-, normo-, and hyperthermia conditions. Sci Rep.

[6] Saeki A, Goto Y, Hata K, Takasago T, Nishioka T, Suga H (2000). Negative inotropism of hyperthermia increases oxygen cost of contractility in canine hearts. Am J Physiol Heart Circ Physiol.

[7] Barja F, Mathison R, Huggel H (1983). Substance P-containing nerve fibers in large peripheral blood vessels of the rat. Cell Tissue Res.

[8] Wharton J, Gulbenkian S, Mulderry PK, Ghatei MA, McGregor GP, Bloom SR, Polak JM (1986). Capsaicin induces a depletion of calcitonin gene-related peptide (CGRP)-immunoreactive nerves in the cardiovascular system of the guinea pig and rat. J Auton Nerv Syst.

[9] Yue Z, Xie J, Yu AS, Stock J, Du J, Yue L (2015). Role of TRP channels in the cardiovascular system. Am J Physiol Heart Circ Physiol.

[10] Hurt CM, Lu Y, Stary CM, Piplani H, Small BA, Urban TJ, Qvit N, Gross GJ, Mochly-Rosen D, Gross ER (2016). Transient receptor potential vanilloid 1 regulates mitochondrial membrane potential and myocardial reperfusion injury. J Am Heart Assoc..

[11] Buckley CL, Stokes AJ (2011). Mice lacking functional TRPV1 are protected from pressure overload cardiac hypertrophy. Channels.

[12] Gao F, Liang Y, Wang X, Lu Z, Li L, Zhu S, Liu D, Yan Z, Zhu Z (2014). TRPV1 activation attenuates high-salt diet-induced cardiac hypertrophy and fibrosis through PPAR-d upregulation. PPAR Res..

[13] Lang H, Li Q, Yu H, Li P, Lu Z, Xiong S, Yang T, Zhao Y, Huang X, Gao P, Zhang H, Shang Q, Liu D, Zhu Z (2015). Activation of TRPV1 attenuates high salt-induced cardiac hypertrophy through improvement of mitochondrial function. Br J Pharmacol.

[14] Wang Q, Ma S, Li D, Zhang Y, Tang B, Qui C, Yang Y, Yang D (2014). Dietary capsaicin ameliorates pressure overload induced cardiac hypertrophy and fibrosis through the transient receptor potential vanilloid type 1. Am J Hypertens.

[15] Mattiazzi A, Mundiña-Weilenmann C, Guoxiang C, Vittone L, Kranias E (2005). Role of phospholamban phosphorylation on Thr17 in cardiac physiological and pathological conditions. Cardiovasc Res.

[16] Dedov VN, Tran VH, Duke CC, Connor M, Christie MJ, Mandadi S, Roufogalis BD (2002). Gingerols: a novel class of vanilloid receptor (VR1) agonists. Br J Pharmacol.

[17] Kobayashi M, Shoji N, Ohizumi Y (1987). Gingerol, a novel cardiotonic agent, activates the Ca^2+^-pumping ATPase in skeletal and cardiac sarcoplasmic reticulum. Biochim Biophys Acta.

[18] Namekata I, Hamaguchi S, Wakasugi Y, Ohhara M, Hirota Y, Tanaka H (2013). Ellagic acid and gingerol, activators of the sarco-endoplasmic reticulum Ca^2+^-ATPase, ameliorate diabetes mellitus-induced diastolic dysfunction in isolated murine ventricular myocardia. Eur J Pharmacol.

[19] Hata Y, Sakamoto T, Hosogi S, Ohe T, Suga H, Takaki M (1998). Effects of thapsigargin and KCl on the O_2_ use of the excised blood-perfused rat heart. J Mol Cell Cardiol.

[20] Hata Y, Sakamoto T, Hosogi S, Ohe T, Suga H, Takaki M (1998). Linear O_2_ use-pressure–volume area relation from curved end-systolic pressure–volume relation of the blood-perfused rat left ventricle. Jpn J Physiol.

[21] Mitsuyama S, Takeshita D, Obata K, Zhang GX, Takaki M (2013). Left ventricular mechanical and energetic changes in long-term isoproterenol-induced hypertrophied hearts of SERCA2a transgenic rats. J Mol Cell Cardiol.

[22] Obata K, Takaki M (2018). Methods for the preparation of an excised, cross-circulated rat heart. Methods Mol Biol.

[23] Takaki M (2004). Left ventricular mechanoenergetics in small animal. Jpn J Physiol.

[24] Tsuji T, Ohga Y, Yoshikawa Y, Sakata S, Abe T, Tabayashi N, Kobayashi S, Kitamura S, Taniguchi S, Suga H, Takaki M (2001). Rat cardiac contractile dysfunction induced by Ca^2+^ overload: possible link to the proteolysis of fodrin. Am J Physiol Heart Circ Physiol.

[25] Yoshikawa Y, Zhang G-X, Obata K, Ohga Y, Matsuyoshi H, Taniguchi S, Takaki M (2010). Cardioprotective effects of a novel calpain inhibitor, SNJ-1945 for reperfusion injury after cardioplegic cardiac arrest. Am J Physiol Heart Circ Physiol.

[26] Matsubara H, Takaki M, Yasuhara S, Araki J, Suga H (1995). Logistic time constant of isovolumic relaxation pressure-time curve in the canine left ventricle. Better alternative to exponential time constant. Circulation..

[27] Takaki M, Akashi T, Ishioka K, Kikuta A, Matsubara H, Yasuhara S, Fujii W, Suga H (1994). Effects of capsaicin on mechanoenergetics of excised cross-circulated canine left ventricle and coronary artery. J Mol Cell Cardiol.

[28] Nakajima-Takenaka C, Sakata S, Kato S, Ohga Y, Murata KY, Taniguchi S, Takaki M (2005). Detrimental effects after dobutamine infusion on rat left ventricular function: mechanical work and energetics. Exp Physiol.

[29] Zhang GX, Obata K, Takeshita D, Mitsuyama S, Nakashima T, Kikuta A, Hirabayashi M, Tomita K, Vetter R, Dillmann WH, Takaki M (2012). Evaluation of left ventricular mechanical work and energetics of normal hearts in SERCA2a transgenic rats. J Physiol Sci.

[30] Docherty RJ, Yeats JC, Piper AS (1997). Capsazepine block of voltage-activated calcium channels in adult rat dorsal root ganglion neurones in culture. Br J Pharmacol.

[31] Liu L, Simon SA (1997). Capsazepine, a vanilloid receptor antagonist, inhibits nicotinic acetylcholine receptors in rat trigeminal ganglia. Neurosci Lett.

[32] Pan HL, Chen SR (2004). Sensing tissue ischemia: another new function for capsaicin receptors?. Circulation.

[33] Watanabe H, Murakami M, Ohba T, Ono K, Ito H (2009). The pathological role of transient receptor potential channels in heart disease. Circ J.

[34] Andrei SR, Sinharoy P, Bratz IN, Damron DS (2016). TRPA1 is functionally co-expressed with TRPV1 in cardiac muscle: co-localization at z-discs, costameres and intercalated discs. Channels (Austin)..

[35] Vostarek F, Svatunkova J, Sedmera D (2016). Acute temperature effects on function of the chick embryonic heart. Acta Physiol (Oxf).

[36] Mattiazzi A, Kranias EG (2014). The role of CaMKII regulation of phospholamban activity in heart disease. Front Pharmacol.

[37] Wang W, Zhu W, Wang S, Yang D, Crow MT, Xiao RP, Cheng H (2004). Sustained β1-adrenergic stimulation modulates cardiac contractility by Ca^2+^/calmodulin kinase signaling pathway. Circ Res.

[38] Lu MJ, Chen YS, Huang HS, Ma MC (2014). Hypoxic preconditioning protects rat hearts against ischemia-reperfusion injury via the arachidonate 12-lipoxygenase/transient receptor potential vanilloid 1 pathway. Basic Res Cardiol.

[39] MacDougall LK, Jones LR, Cohen P (1991). Identification of the major protein phosphatases in mammalian cardiac muscle which dephosphorylate phospholamban. Eur J Biochem.

[40] Lotteau S, Ducreux S, Romestaing C, Legrand C, van Coppenolle F (2013). Characterization of functional TRPV1 channels in the sarcoplasmic reticulum of mouse skeletal muscle. PLoS One..

[41] Dogan MD, Patel S, Rudaya AY, Steiner AA, Székely M, Romanovsky AA (2004). Lipopolysaccharide fever is initiated via a capsaicin-sensitive mechanism independent of the subtype-1 vanilloid receptor. Br J Pharmacol.

[42] Fujimoto S, Mori M, Tsushima H, Kunimatsu M (2006). Capsaicin induces the relaxation of the Guinea-Pig Ileum, which is not mediated by TRPV1, and is Capsazepine-Insensitive. Eur J Pharmacol.

[43] Mahmoud ME, Shimizu Y, Shiina T, Nikami H, Dosoky RM, Ahmed MM, Takewaki T (2007). Involvement of a capsaicin-sensitive TRPV1-independent mechanism in lipopolysaccharide-induced fever in chickens. Comp Biochem Physiol A.

[44] Sharma SK, Vij AS, Sharma M (2013). Mechanisms and clinical uses of capsaicin. Eur J Pharmacol.

[45] Thakre PP, Bellingham MC (2019). Capsaicin causes robust reduction in glycinergic transmission to rat hypoglossal motor neurons via a TRPV1-independent mechanism. J Neurophysiol.

[46] Bao Z, Dai X, Wang P, Tao Y, Chai D (2019). Capsaicin induces cytotoxicity in human osteosarcoma MG63 cells through TRPV1-dependent and -independent pathways. Cell Cycle.

