# Supplementary material for: Mechanism underlying the negative inotropic effect in rat left ventricle in hyperthermia: the role of TRPV1
Source: J Physiol Sci. 2020 Feb 5;70:4. doi: 10.1186/s12576-020-00734-5 (PMC7002332; doi:10.1186/s12576-020-00734-5)

**Additional methods**

**Experimental animals**

All experiments were in accordance with the Guide for the Care and Use of Laboratory Animals published by the National Institutes of Health (NIH Publication No. 85-23, Revised 1996), and the protocols were approved by the Gifu University Institutional Animal Care and Use Committee. Male Wistar rats purchased from SLC Japan Inc. (Hamamatsu, Japan).

**Surgical preparation**

In the present study, we used the excised cross-circulated rat heart preparation as previously published (1-6) (supplemental Fig.1). In each experiment, we used 3 rats: one rat was used as a heart donor, and 2 retired breeder male Wistar rats weighing 510 ± 49.5 g were used as blood supplier weighing 417 ± 65.0 g and metabolic supporter rats, respectively. These rats were anesthetized with pentobarbital sodium (50 mg/kg ip) and intubated. All rats were heparinized (1,000 units iv). After a median sternotomy, the blood was extracted via the left ventricular (LV) apex of a blood supplier rat. This blood was used for priming the cross-circulation tubing. The beating heart was excised without interruption of coronary perfusion and supported by cross circulation with the metabolic supporter rat (supplemental Fig.1). In brief, the bilateral common carotid arteries and right external jugular vein of the metabolic supporter rat were cannulated and connected to the brachiocephalic artery and right ventricle (RV) via the superior vena cava in the heart donor rat, respectively. The beating heart supported by cross-circulation was subsequently excised from the chest of the heart donor rat and was maintained at 37°C. A thin latex balloon (balloon material volume, 0.08 ml) fitted into the LV space was connected to a pressure transducer (Life Kit DX 312, Nihon Kohden; Tokyo, Japan) and a 0.5-ml precision glass syringe with fine scales (minimum scale, 0.005 ml). Thus, LV volume (LVV) was changed and measured by adjusting the intra-balloon water volume with the syringe in 0.025-ml steps between 0.08 and 0.23 ml (balloon material volume + intra-balloon water volume). The LV epicardial electrocardiogram was recorded, and the heart rate was constantly maintained at 300-beats per minute (bpm) by electrical pacing of the right atrium. The systemic arterial blood pressure (BP) of the supporter rat served as the coronary perfusion pressure (approximately 100 mmHg). Arterial pH, PO_2_, and PCO_2_ of the supporter rat were maintained within their physiological ranges with supplemental O_2_ and sodium bicarbonate. Blood lactate was measured with Lactate Pro (Arkray, Kyoto, Japan). We confirmed no increase in the mean values of arteriovenous lactate difference at the maximum LVV loading (the maximum O_2_ demand). The myocardial temperature was changed from 37°C (normothermic condition) to 42°C (hyperthermic condition) with ThermoClamp™-1 temperature controller system (inline-type, AutoMate Scientific, Inc., CA) for pre-incubation 30 min before data sampling. The anesthetic level of the metabolic supporter rat was maintained at a constant level via additional continuous infusion of pentobarbital sodium at 7.5 mg/h by monitoring the systemic arterial pressure and heart rate. In each steady state, all data were measured and sampled at 1 kHz for 5–10 s and averaged using a PowerLab unit and LabChart software (AD Instruments, Bella Vista, NSW, Australia).

**Oxygen consumption**

Myocardial VO_2_ was obtained as the product of the coronary blood flow (CBF) and coronary arteriovenous O_2_ content difference (AVO_2_D) (supplemental Fig.1). Total CBF was continuously measured with an ultrasonic flowmeter (T410, Nihon Nipro-Transonic Japan, Saitama, Japan) placed in the middle of the coronary venous drainage tubing from the RV. LV thebesian flow was negligible. The AVO_2_D was continuously measured by passing all arterial and venous cross-circulation blood through the two cuvettes of a custom-made AVO_2_D analyzer (PWA 200S, Shoe Technica; Chiba, Japan).

**Experimental protocol**

**· Volume-loading run: vol-run**

LVV was changing and measured by adjusting the intra-balloon water volume with the syringe in 0.025-ml steps between 0.08 ml and 0.23 ml (5 to 6 different volumes) (volume-loading run: vol-run) in presence or absence of capsazepine (CPZ, final concentration, 1–2 µg/ml) or capsaicin, (Cap, final concentration, 20–500 ng/ml) during 37°C or 42°C (supplemental Fig.1). In every vol-run, a steady state, where LV pressure (LVP), AVO_2_D, and CBF were stable, was reached 2–3 min after changing LVV.

**· Inotropism run: ino-run**

After the vol-run, a Cap inotropic run (Cap ino-run) was performed during 62.5 μg/ml Cap solution infusion with a micro-syringe pump during 37°C. LVV was fixed at midrange LVV (mLVV) (0.16 ml = 0.08 ml [V_0_] plus 0.08 ml [a half value between the minimum and maximum water volume infused into the balloon]). At first, LVP, VO_2_, and PVA data were obtained as zero Cap before the infusion. The intracoronary infusion of 62.5 μg/ml Cap solution was started at 1.0 μl/min and the infusion rate of Cap was gradually increased from 1.0 to 20.0 μl/min. LVP, VO_2_, and PVA data at each concentration were obtained until a decrease in end-systolic pressure (ESP) or arrhythmia.

**· KCl-induced cardiac arrest**

Cardiac arrest was induced by infusing KCl (0.5 mol/l) into the coronary perfusion tubing at a constant rate (5−10 ml/h) with syringe pump (supplemental Fig.1) to measure the basal metabolic O_2_ consumption, which was adjusted to abolish electrical excitation under monitoring ventricular electrocardiograms but not to generate any KCl-induced constrictions of coronary vessels. VO_2_ data were obtained by minimal volume loading to avoid volume-loading effects, if any, on VO_2_ data.

**Data analysis**

**· ESPVRs, EDPVRs, systolic pressure-volume area (PVA), V_0_, and mLVV**

As shown previously (2-6), we obtained the best-fit end-systolic pressure-volume relation (ESPVR) and end-diastolic pressure-volume relation (EDPVR) from 5 to 6 different pressure-volume data with the two different exponential functions by means of the least-squares method (Delta-Graph, Red Rock Software, Inc. UT) on a personal computer (supplemental Fig.2A). We attempted to fit experimentally obtained LV pressure-volume (P-V) data using the following equations to obtain ESPVRs and EDPVRs.

1. Pes = A { 1 – exp [ –B (V – V_0_) ] }
2. Ped = A’{ exp [ B’ (V – V_u_) ] – 1 }

where Pes and Ped are end-systolic and end-diastolic peak isovolumic pressure, respectively, V is isovolumic volume, and V_0_ and V_u_ are the volume intercepts of the nonlinear curves best fitted to the end-systolic and end-diastolic P-V points, respectively. A, A’, B, B’, V_0_, and V_u_ are fitting parameters (2-6). The systolic pressure-volume area (PVA) is defined as the PVA circumscribed by the curvilinear best-fit ESPVRs, EDPVRs, and the systolic portions of the ventricular P-V trajectories at any LVVs (supplemental Fig.2A). The area under the best-fit EDPVR was subtracted from the area under the best-fit ESPVR to obtain the net PVA. Systolic unstressed volume (V_0_) was determined by filling the balloon to the level where peak isovolumic pressure and hence PVA were zero. The sum of intra-balloon water volume and balloon material volume was used as an initial estimate of V_0_. This procedure was repeated during different LVV-loading runs. V_0_ was then finally determined as the volume-axis intercept of the best-fit ESPVR. In the present study, we calculated PVA at mLVV to assess LV mechanoenergetics based on our previous studies (2-5). A mLVV corresponded to 0.16 ml [= (0.23 – V_0_)/2 + V_0_] (a half value between the minimum and maximum water volume infused into the balloon). Finally, LVV, PVA, V_0_, and mLVV were normalized by LV mass to 1 g.

**· VO_2_-PVA relation**

As shown previously (2-6), the VO_2_-PVA relation was linear in the rat LV (supplemental Fig.2B). Its slope represents the O_2_ cost of PVA (contractile efficiency), and its VO_2_ intercept represents PVA-independent VO_2_. The PVA-independent VO_2_ is composed of O_2_ consumption for Ca^2+^ handling in E-C coupling and for basal metabolism (supplemental Fig.2B). The RV was kept collapsed by continuous hydrostatic drainage of the coronary venous return so that the RV PVA and hence PVA-dependent VO_2_ were assumed to be negligible. The RV component of PVA independent VO_2_ was calculated by multiplying biventricular PVA-independent VO_2_ in each contractile state with the ratio of RV weight divided by the sum of RV and LV weights. The RV PVA-independent VO_2_ was subtracted from the total VO_2_ to yield LV VO_2_. The LV (including the septum) and RV were weighed for normalization of LVV.

**References**

1. **Obata K, Takaki M.** Methods for the preparation of an excised, cross-circulated rat heart. ***Methods Mol Biol*** 1816: 117-132, 2018.
2. **Mitsuyama S, Takeshita D, Obata K, Zhang GX, Takaki M.** Left ventricular mechanical and energetic changes in long-term isoproterenol-induced hypertrophied hearts of SERCA2a transgenic rats. ***J Mol Cell Cardiol*** 59: 95-106, 2013.
3. **Hata Y, Sakamoto T, Hosogi S, Ohe T, Suga H, Takaki M.** Linear O_2_ use-pressure-volume area relation from curved end-systolic pressure-volume relation of the blood-perfused rat left ventricle. ***Jpn J Physiol*** 48: 197-204, 1998.
4. **Hata Y, Sakamoto T, Hosogi S, Ohe T, Suga H, Takaki M.** Effects of thapsigargin and KCl on the O_2_ use of the excised blood-perfused rat heart. ***J Mol Cell Cardiol*** 30: 2137-2143, 1998.
5. **Yoshikawa Y, Zhang GX, Obata K, Ohga Y, Matsuyoshi H, Taniguchi S, Takaki M.** Cardioprotective effects of a novel calpain inhibitor, SNJ-1945 for reperfusion injury after cardioplegic cardiac arrest. ***Am J Physiol Heart Circ Physiol*** 298: H643-H651, 2010.
6. **Takaki M.** Left ventricular mechanoenergetics in small animal. ***Jpn J Physiol*** 54: 175-207, 2004.

**Additional** Fig. S1.

Schematic illustration of experimental setting for the excised blood-perfused rat heart


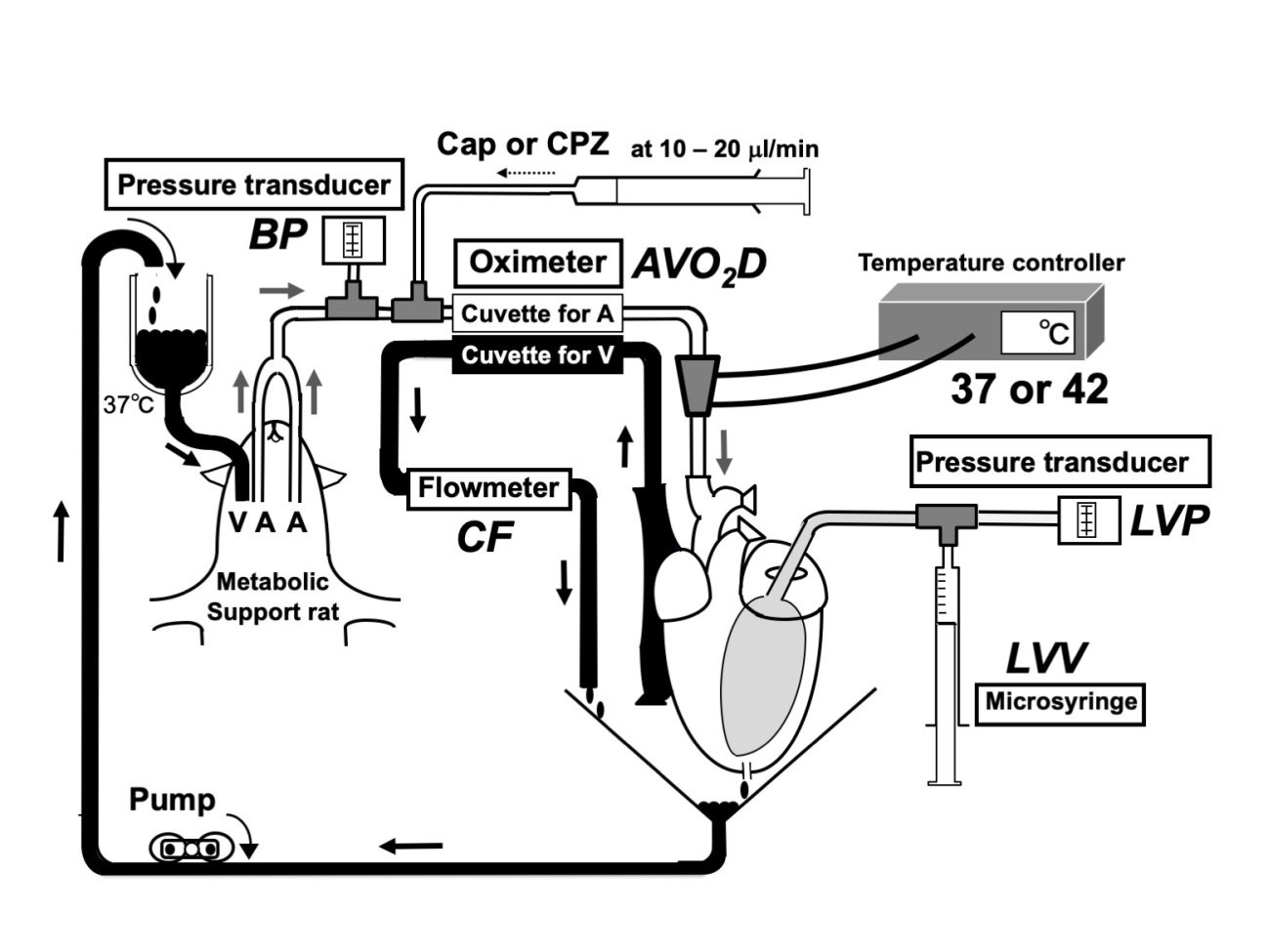


**Additional** Fig. S2.

Schematic illustration of framework of ESPVR-VO_2_-PVA


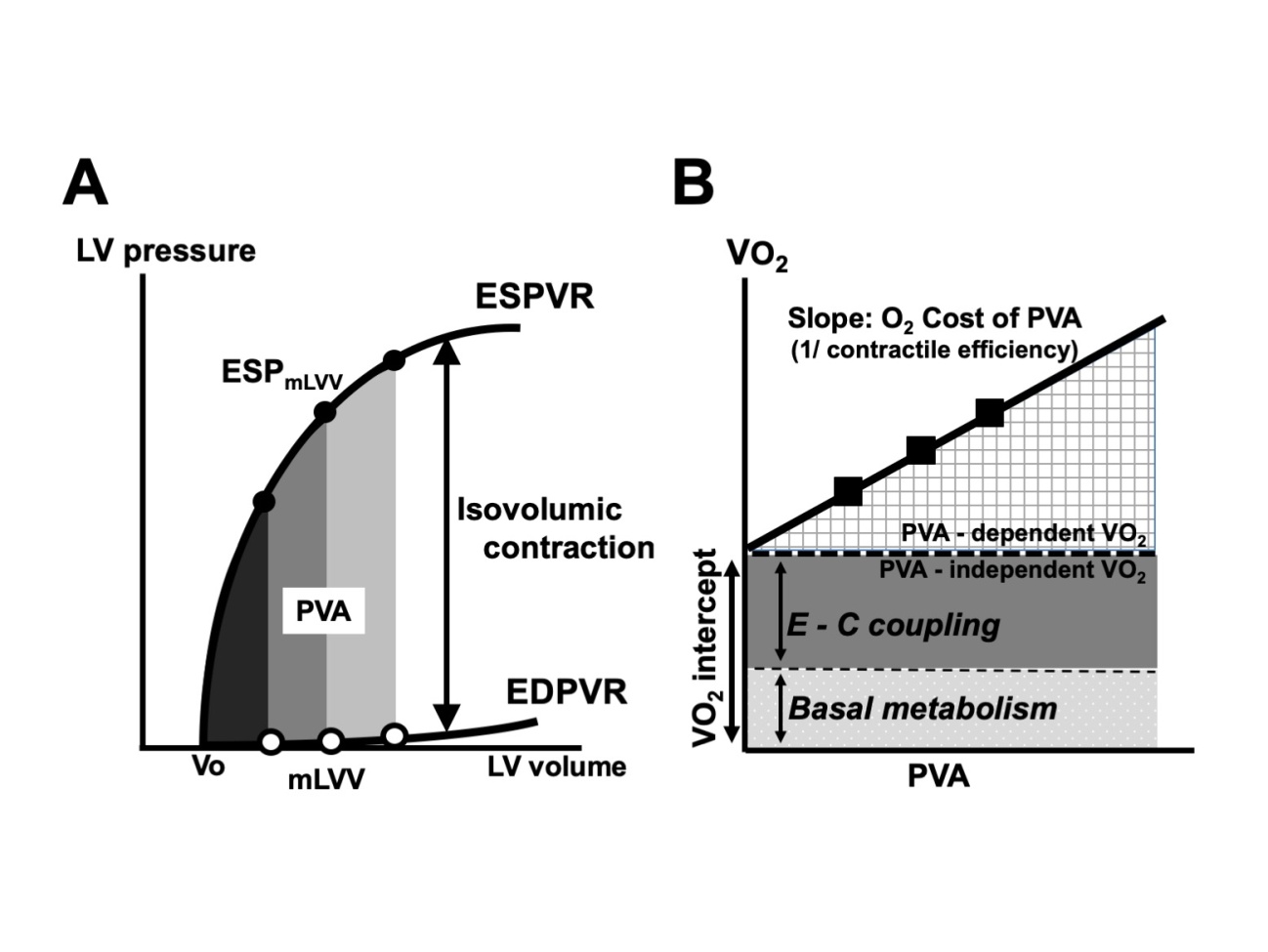

Supplement: Supplementary file 1 — Additional file 1. Supplemental methods for the excised cross-circulated rat heart model and the data analysis. Figure S1. Schematic illustration of experimental setting for the excised blood-perfused rat heart. Figure S2. Schematic illustration of framework of ESPVR-VO2-PVA. [file 12576_2020_734_MOESM1_ESM.docx]
